# Effect of prenatal EPA and DHA on maternal and umbilical cord blood cytokines

**DOI:** 10.1186/s12884-018-1899-6

**Published:** 2018-06-26

**Authors:** Ellen L. Mozurkewich, Deborah R. Berman, Anjel Vahratian, Chelsea M. Clinton, Vivian C. Romero, Julie L. Chilimigras, Delia Vazquez, Clifford Qualls, Zora Djuric

**Affiliations:** 1 Department of Obstetrics and Gynecology, University of New Mexico School of Medicine, MSC 10 5580, 1 University of New Mexico, Albuquerque, NM 87131 USA; 2 Obstetrics and Gynecology, University of Michigan, Ann Arbor, MI USA; 3 Obstetrics and Gynecology, Duke University, Durham, NC USA; 4 Obstetrics and Gynecology, Spectrum Health Maternal Fetal Medicine, Grand Rapids, MI/Michigan State University College of Human Medicine, East Lansing, MI USA; 5 Department of Pediatrics, University of Michigan, Ann Arbor, MI USA; 6 Clinical and Translational Center, University of New Mexico, Albuquerque, NM USA; 7 Family Medicine, University of Michigan, Ann Arbor, MI USA

**Keywords:** Cytokines, EPA, DHA, Umbilical cord blood

## Abstract

**Background:**

Investigators have hypothesized that omega-3 fatty acid supplementation may modulate the immune response. However, available evidence is conflicting. We performed this study to investigate the effect of prenatal eicosapentaenoic acid (EPA)- and docosahexaenoic acid (DHA)-rich fish oil supplementation on maternal and fetal cytokine production.

**Methods:**

This study is a secondary analysis of a randomized controlled trial designed to assess whether prenatal EPA- or DHA-rich fish oil supplementation would prevent perinatal depressive symptoms among women at risk. Enrolled participants received EPA-rich fish oil (1060 mg EPA plus 274 mg DHA), DHA-rich fish oil (900 mg DHA plus 180 mg EPA) or soy oil placebo. Maternal venous blood was collected at enrollment (12–20 weeks gestation) and after supplementation (34–36 weeks gestation). Umbilical cord blood was collected at delivery. We analyzed stored plasma specimens for 16 human cytokines using multiplex immunoassays. Maternal and cord blood cytokine levels were compared among the treatment groups. Associations of serum DHA and EPA with maternal and cord blood cytokines were explored via regression analysis.

**Results:**

We enrolled 126 women, of whom 118 completed the trial. Prenatal supplementation with EPA-rich fish oil significantly lowered maternal IL6, IL15, and TNFα concentrations. However, supplementation with DHA-rich fish oil had no significant effect on maternal cytokine profiles. Maternal serum DHA fraction was significantly associated with IL1α, and maternal serum DHA and EPA fractions were significantly associated with IL 10 concentrations after supplementation. Compared with placebo, supplementation with EPA- or DHA-rich fish oils had no significant effect on cord blood cytokine concentrations.

**Conclusions:**

Prenatal supplementation with EPA-rich fish oil significantly reduced levels of several inflammatory cytokines in maternal plasma, while prenatal DHA-rich fish oil had no significant effect on cytokine concentrations. Supplementation with EPA- and DHA- rich fish oil had no significant effect on umbilical cord blood cytokine concentrations.

**Trial registration:**

Clinical Trial Registration: registration number NCT00711971 7/7/2008.

## Background

Inflammation has been associated with many complications of pregnancy, including perinatal depression, gestational diabetes, preterm delivery and preeclampsia [1, 2]. For example, a recent case-control study found that elevated concentrations of TNF- α in mid-pregnancy were significantly associated with diagnoses of preeclampsia later in pregnancy, [3] Similarly increases in circulating IL-1-β, IL-6, IL-18 and TNF have been demonstrated in women with GDM complicating pregnancy compared with controls [2]. Likewise, a recent case-control study of inflammatory markers among subjects with perinatal depression found that a diagnosis of major depressive disorder was significantly related to increased concentrations of TNFα [4].

The Western diet has been postulated to be one important cause of inflammation [5, 6]. Some investigators have related the increase in inflammatory disorders in the developed world to an imbalance in dietary intake in omega-6 to omega-3 fat compared to historical human diet [7]. Dietary supplementation with omega-3 fatty acids has therefore been proposed as a mechanism to potentially reduce inflammation [7].

Omega-3 fatty acids, both in food and through supplementation with fish or algal oil, have long been believed to limit or modulate the inflammatory response through modulation of cytokine production. Two early omega-3 fatty acid supplementation studies in healthy adults using greater than 2.4 g of EPA plus DHA daily reported significantly reduced blood cytokines TNFα, IL1β, and IL6 [7–9]. However, a systematic review of randomized trials using DHA and EPA supplementation among healthy adults found no consistent effect of supplementation on blood cytokines [10]. The majority of studies investigating the effect of dietary supplementation on cytokines have measured cytokines after endotoxin stimulation of cells, whereas, more recent studies have measured circulating soluble cytokines using multiplex immunoassays [11]. It is not known whether fish oil supplementation during pregnancy modulates maternal cytokine production or whether supplementation alters cytokine concentrations in the umbilical cord blood.

This study is a secondary analysis of a double-blind randomized control trial carried out to test the hypothesis that EPA- or DHA-rich fish oil supplementation would reduce perinatal depressive symptoms. A secondary aim of the study was to determine the effect of prenatal EPA- and DHA- risk fish oil supplementation on maternal cytokine profiles as well as on cytokines in umbilical cord blood [12]. We hypothesized that prenatal EPA- and DHA-rich fish oil supplementation would reduce concentrations of the major pro-inflammatory cytokines IL-β, IL-6, and TNF-α in both maternal and umbilical cord plasma.

## Methods

This study is a planned secondary analysis of plasma samples that were collected as part of a prospective, double-blind randomized controlled trial of fish oil supplementation for prevention of depressive symptoms among pregnant women [12, 13]. To be eligible to participate in the parent study, women had to be between 12 and 20 weeks gestation in a singleton pregnancy and to be at risk for depression based on an Edinburgh Postnatal Scale Score between 9 and 19 or a past history of depression. Potential subjects consuming more than two fish meals per week were not eligible to participate. Subjects were recruited from the antenatal clinics of the University of Michigan Medical Center (Ann Arbor, MI) and St. Joseph Mercy Health System (Ypsilanti, MI). The overall details of the trial design have been previously reported [12, 13].

The sample size for the study was determined based on the expected Beck Depression Inventory scores among women at risk for depression. The parent study was designed to detect a 50% reduction in the primary outcome for the trial, the Beck Depression Inventory score at 6 weeks postpartum [12, 13]. Maternal and umbilical cord cytokine levels were a pre-specified secondary outcome of the study [12]. We used a random number table maintained in the University of Michigan Investigational Drug Pharmacy for random allocation. Full details of the randomized, double-blinded study design as well as the primary and clinical outcomes have been previously reported [12, 13].

There were 126 subjects enrolled in the parent study between October 2008 and May 2011. They were randomly assigned to receive EPA rich fish oil (1060 mg EPA plus 274 mg DHA), DHA rich fish oil (900 mg DHA plus 180 mg EPA), or soy oil placebo and were followed longitudinally through pregnancy from their enrollment at 12–20 weeks’ gestation until 6 weeks postpartum. Maternal blood was drawn at enrollment and again at 34–36 weeks gestation. The minimum supplementation time was 14 weeks. Because of the double-dummy design of the trial, all subjects received some placebo capsules. Umbilical cord blood was obtained at delivery.

**Fig. 1:**
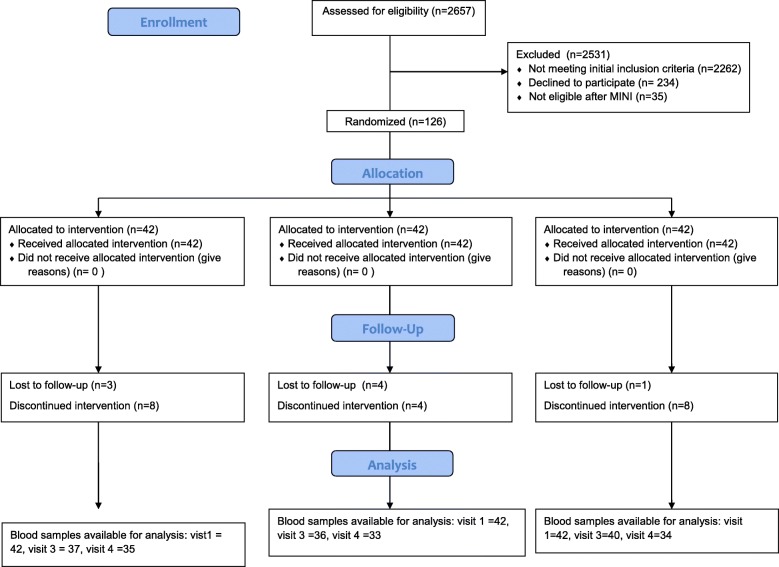
Consort 2010 Flow Diagram

During the course of the study, eight subjects dropped out and 118 subjects completed the study. There were 126 maternal samples available for cytokine analysis at enrollment and 113 maternal samples available for analysis after supplementation (34–36 weeks). There were 102 cord blood samples available for analysis of cytokine profiles. The flow diagram of participant enrollment and sample availability is depicted Fig. 1.

### Sample collection and biochemical assessment

We obtained venous blood after a four hour fast. Fetal cord blood was collected at delivery. Cord blood was obtained after severance of the umbilical cord by passive flow of mixed arterial and venous blood into EDTA-containing tubes. All blood samples were processed by centrifugation within 12 h of collection and the resultant plasma was stored at − 80 °C [12, 13]. Multiplex immunoassays for cytokines IL-1α, IL-1β, IL-2, IL-4,IL-5,IL-6,IL-8, IL-10, IL-12p70, IL-13, IL-15, IL-17, IFNγ,MCP-1, TNFα, and TNFβ were performed on plasma samples using the Q-Plex™ Human Cytokine - Screen IR (16-plex), a fully quantitative ELISA-based Infrared (IR) assay system (Quansys Biosciences, Logan, UT).

Serum EPA and DHA, had been previously assayed from sample collection of maternal and cord blood into red top non-additive tubes at the same time points, with DHA and EPA being evaluated as a measure of compliance [13]. As we have previously reported, supplementation with the DHA-rich fish oil significantly increased serum DHA levels in maternal and cord blood. Similarly, supplementation with EPA-rich fish oil significantly increased maternal serum EPA fraction but not cord blood EPA. These variables were available from prior study to evaluate as predictors of maternal and cord blood plasma cytokine levels [13].

### Ethics

The procedures followed were approved and conducted in accordance with the institutional review boards of the University of Michigan Medical Center, Ann Arbor, MI, St. Joseph Mercy Hospital in Ypsilanti, MI, and The University of New Mexico Health Sciences Center Human Research Protections Office. All subjects gave written informed consent to participate in the study. The trial was registered on 7/7/2008 at clinicaltrials.gov: NCT00711971 under the title: “Does Fish Oil Prevent Depression in Pregnancy and Postpartum”.

### Statistics

For a simple post-hoc power analysis, we used our change in maternal plasma IL-6 at visit 3 (34–36 weeks) between EPA-rich fish oil and placebo. For our sample size of 37 in the EPA group and 40 in the placebo group, we could detect a group difference in the change in IL-6 of 80 pg/ml with 80% power and a significance of 0.05. This power analysis was based on the standard deviations (94 pg/ml and 176 pg/ml) that we observed in these two groups. Although this study’s sample size was determined in order to evaluate a different primary outcome measure (the Beck Depression Inventory score at 6 weeks’ postpartum), it had adequate power (80%) to detect a group difference between the change in maternal IL-6 after supplementation compared to placebo.

We computed median and interquartile range values for the all of the cytokines at each of the time points. If the value for a sample was reported as “below detectable limits”, the value of half the lower limit of detection for the cytokine was used for the purposes of subsequent comparisons, a technique known as “single value imputation” [14]. Cytokine concentrations were log-transformed in order to symmetrize the skewed distributions. All analyses were according to intention to treat principles. For all ANCOVA analyses that evaluated the effect of DHA- and EPA- rich fish oil supplementation on maternal cytokines, we adjusted for the baseline values of fatty acids (EPA and DHA fractions) and cytokines by subtraction of visit 1 values from visit 3 values. Where there was a significant treatment effect, post hoc multiple comparisons between the treatments was done by Fisher’s Least Significant Difference method.

In addition, we adjusted for maternal BMI at enrollment (visit 1). Because of the potential correlation of allocated treatment group with EPA and DHA concentrations, the model was analyzed with and without each of these variables. Pearson’s correlation coefficients were computed to describe the relationship between serum EPA and DHA fractions, maternal BMI and the cytokines under study.

We performed ANCOVA analyses to describe the relationship between the group of allocation and cytokines in the umbilical cord blood. All analyses were adjusted for exposure to labor (regardless of mode of delivery), gestational age at delivery, birth weight, as well as DHA and EPA concentrations in umbilical cord blood. Analyses were carried out with all variables in the model as well as with correlated variables removed. Pearson correlation coefficients were constructed to evaluated the relationship between each of the variables in the model and the cytokines under study in order to assess the changes in cytokines between enrollment and late pregnancy pooled across groups, we computed the differences between visits 3 and visit 1 and compared to no change using one sample t tests.

## Results

### Effect of fish oil supplementation on maternal cytokines

There were 126 samples available for maternal cytokine analyses at enrollment and 113 samples available after supplementation. The median and interquartile ranges of the maternal cytokines at two time points are presented in Table 1.

**Table 1:** Maternal cytokine values before and after supplementation

	LLD		Enrollment 1 *N* = 126		Post-supplementation *N* = 113	
			median	IQR	median	IQR
IL1α	6.664	EPA	6.66	(6.66,11.3)	6.66	(6.66,6.66)
		DHA	6.66	(6.66,15.4)	6.66	(6.66,6.66)
		Placebo	6.66	(6.66,6.66)	6.66	(6.66,10.6)
IL1β	7.806	EPA	13.2	(7.81,48.5)	15.7	(7.81,68.7)
		DHA	7.81	(7.81,48)	16.3	(7.81,63.6)
		Placebo	9.65	(7.81,46.7)	23	(7.81,74.2)
IL2	2.584	EPA	26.7	(2.58,64.1)	23.1	(6.4,57.9)
		DHA	27.9	(6.1,71.4)	15.5	(2.58,48.1)
		Placebo	30.5	(2.58,61.8)	28.2	(2.58,61.7)
IL4	0.776	EPA	0.776	(0.78,4.7)	0.776	(0.776,2.5)
		DHA	0.776	(0.78,3.8)	0.776	(0.776,6.2)
		Placebo	0.776	(0.78,3.7)	0.776	(0.776,2.15)
IL5	35.05	EPA	35.1	(35.1106)	35.1	(35.1107)
		DHA	35.1	(35.1,48.4)	35.1	(35.1128)
		Placebo	35.1	(35.1,67.9)	35.1	(35.1,95.2)
IL6	18.466	EPA	18.5	(18.5,41.8)	18.5	(18.5,23.6)
		DHA	18.5	(18.5,18.5)	18.5	(18.5,50.2)
		Placebo	18.5	(18.5,18.5)	18.5	(18.5,36.8)
IL8	9.894	EPA	9.89	(9.89,18.7)	9.89	(9.89,38.4)
		DHA	9.89	(9.89,12)	9.89	(9.89,24.3)
		Placebo	9.89	(9.89,9.89)	9.89	(9.89,26.9)
IL10	2.94	EPA	2.94	(2.94,48.3)	2.94	(2.94,32.5)
		DHA	2.94	(2.94,37.3)	2.94	(2.94,57.4)
		Placebo	2.94	(2.94,43.3)	2.94	(2.94,36.0)
IL12p70	45.458	EPA	45.5	(45.5,99.3)	45.5	(45.5108)
		DHA	45.5	(45.5,71.7)	45.5	(45.5,76)
		Placebo	45.5	(45.5154)	45.5	(45.5,94.1)
IL13	12.608	EPA	12.6	(12.6,55.3)	12.6	(12.6,29.2)
		DHA	12.6	(12.6,58.6)	12.6	(12.6,42.8)
		Placebo	12.6	(12.6,33.1)	12.6	(12.6,44)
IL15	7.734	EPA	7.73	(7.73,29.2)	7.73	(7.73,7.73)
		DHA	7.73	(7.73,7.73)	7.73	(7.73,13.2)
		Placebo	7.73	(7.73,7.73)	7.73	(7.73,25)
IL17	45.586	EPA	45.6	(45.6,49.7)	45.6	(45.6,45.6)
		DHA	45.6	(45.6,45.6)	45.6	(45.6,45.6)
		Placebo	45.6	(45.6,86.9)	45.6	(45.6,59.8)
IFNγ	58.506	EPA	58.5	(58.5104)	58.5	(58.5294)
		DHA	58.5	(58.5156)	58.5	(58.5206)
		Placebo	58.5	(58.5142)	58.5	(58.5159)
MPC1	5.686	EPA	125	(83.6224)	139	(82.6212)
		DHA	118	(75.9228)	107	(60.2183)
		Placebo	138	(88,183)	129	(75.1171)
TNFα	15.146	EPA	15.1	(15.1114)	15.1	(15.1,46.9)
		DHA	15.1	(15.1,15.1)	15.1	(15.1,41.5)
		Placebo	15.1	(15.1,48.2)	15.1	(15.1,58.1)
TNFβ	4.956	EPA	4.96	(4.96,10.9)	4.96	(4.96,4.96)
		DHA	4.96	(4.96,10.5)	4.96	(4.96,15.8)
		Placebo	4.96	(4.96,16.5)	4.96	(4.96,4.96)

All values in pg/ml

IQR = inter quartile range

LLD = lower limit of detection

Range of values below detectable limits from 2.5 to 83.3%

Numbers of samples per group:

EPA = 37

DHA = 36

Placebo = 40

There was no statistically significant change in the concentrations of the cytokines under study between early and late pregnancy. We modeled the change in the cytokines of interest between enrollment (12–20 weeks) and after supplementation (34–36 weeks) with adjustment for body mass index (BMI) at enrollment, DHA, EPA, and group allocation. EPA-rich fish oil treatment decreased plasma concentrations of IL6 (*P* = 0.02), IL 15 (*P* = 0.03), and TNF α (*P =* 0.03) after adjustment for baseline cytokine concentrations, and BMI. Treatment group was not significantly related to the change in IL 1 β, IL2, IL5, IL8, IL12P70, IL17, IFN γ, or MCP1 on post hoc multiple comparisons. We found no significant effect of the DHA-rich fish oil treatment on cytokine concentrations, when compared with placebo.

Our model evaluated the relationship of the variables in the model with the cytokines of interest. We found a positive association between maternal serum DHA fraction, adjusted for DHA fraction at baseline, with IL 1 α (*P* = 0.05) and with IL10, a cytokine with both pro- and anti-inflammatory properties (*P =* 0.05) [15]. By contrast EPA fraction was inversely (negatively) associated with IL10 concentrations (*P* = 0.04). BMI at entry to the study was inversely associated with TNF beta (*P =* 0.04). These relationships are summarized in Table 2. These findings were not altered when the correlated variable of study group allocation as well as EPA and DHA fraction were analyzed separately in the model.

**Table 2:** Variables predictive of maternal cytokine concentrations

Variable	Cytokine	Slope	Standard error +/−	Significance
EPA-rich fish oil group	IL6	−76.3	32.2	0.02
EPA-rich fish oil group	IL15	−23.8	10.9	0.03
EPA-rich fish oil group	TNFα	− 141.1	62.6	0.03
DHA fraction	IL1α	4.64	2.33	0.05
DHA fraction	IL10	4.82	2.36	0.04
EPA fraction	IL10	−18.8	8.42	0.03
BMI at entry	TNFβ	−0.64	0.32	0.05

All analyses were adjusted for the following variables: Baseline concentrations of each cytokine, maternal BMI, maternal serum EPA fraction adjusted for baseline and DHA fraction adjusted for study baseline

For fetal (cord blood) cytokines, there were 102 cord blood samples available for analysis. The median and interquartile ranges of the cord blood cytokines are represented in Table 3. There were no significant differences in cord blood cytokines according to treatment group. Cord blood DHA fraction was negatively correlated with cord blood IL 1β (*P* = 0.03), a relationship that however did not persist after adjusting for labor and gestational age at delivery. There were no other significant associations between EPA or DHA to concentrations of any of the cytokines that we studied. Of other variables included in the model, we found that gestational age was positively correlated with IL8 (*P* = 0.05). When serum EPA and DHA fraction were excluded from the model, exposure to labor was positively correlated with IL10 (*P* = 0.04). There were no other significant relationships demonstrated.

**Table 3:** Cord blood cytokine concentrations

	LLD		median	IQR
IL1α	6.664	EPA	6.66	(6.66,13.9)
		DHA	6.66	(6.66,6.66)
		Placebo	6.66	(6.66,13.1)
IL1β	7.806	EPA	7.81	(7.81,60.1)
		DHA	7.81	(7.81,47.4)
		Placebo	25.9	(7.81,72.7)
IL2	2.584	EPA	33.7	(2.58,81.7)
		DHA	39.0	(21.8,69.2)
		Placebo	32.5	(13.4,52.1)
IL4	0.776	EPA	0.776	(0.78,5.2)
		DHA	0.776	(0.78,4)
		Placebo	1.80	(0.78,5.4)
IL5	35.05	EPA	35.1	(35.1114)
		DHA	35.1	(35.1,84.8)
		Placebo	35.1	(35.1124)
IL6	18.466	EPA	20.1	(18.5,65.1)
		DHA	23.2	(18.5,66)
		Placebo	18.5	(18.5,40.7)
IL8	9.894	EPA	35.7	(9.89,83)
		DHA	10.5	(9.89,55.9)
		Placebo	12.3	(9.89,75.5)
IL10	2.94	EPA	2.94	(2.94,41.7)
		DHA	2.94	(2.94,36.3)
		Placebo	2.94	(2.94,56.5)
IL12p70	45.458	EPA	45.5	(45.5198)
		DHA	45.5	(45.5,45.5)
		Placebo	45.5	(45.5150)
IL13	12.608	EPA	12.6	(12.6,67.3)
		DHA	12.6	(12.6,36.3)
		Placebo	12.6	(12.6,54.9)
IL15	7.734	EPA	7.73	(7.73,20.1)
		DHA	7.73	(7.73,7.73)
		Placebo	7.73	(7.73,7.73)
IL17	45.586	EPA	45.6	(45.6,62.6)
		DHA	45.6	(45.6,45.6)
		Placebo	45.6	(45.6,65.8)
IFNγ	58.506	EPA	58.5	(58.5188)
		DHA	58.5	(58.5193)
		Placebo	58.5	(58.5200)
MPC1	5.686	EPA	309	(192,509)
		DHA	277	(188,473)
		Placebo	288	(171,404)
TNFα	15.146	EPA	15.1	(15.1101)
		DHA	15.1	(15.1,78)
		Placebo	15.1	(15.1,30.9)
TNFβ	4.956	EPA	4.96	(4.96,19.4)
		DHA	4.96	(4.96,4.96)
		Placebo	4.96	(4.96,12.9)

All values in pg/ml

IQR = interquartile range

LLD = lower limit of detection

Range of values below detectable limits from 2.9 to 84.8%

Number of samples per group:

EPA = 35

DHA = 33

Placebo = 34

## Discussion

We found that some maternal plasma cytokine concentrations may be significantly modulated by dietary supplementation during pregnancy. Our study found that supplementation with EPA-rich fish oil in particular resulted in reduced concentrations of IL6 and TNFα, inflammatory cytokines that have been associated with major depression in other studies [16]. These findings are similar to the results of a randomized control trial of supplementation with 2 g/day of omega-3 fatty acids among obese pregnant women. In that trial, prenatal supplementation was found to reduce inflammatory markers in maternal adipose tissue and in the placenta [17]. This finding is in contrast with previous observations among healthy non-pregnant adults in which supplementation with fish oil up to 3.5 g/day had no significant effect on cytokine profiles [10]. The reason for this finding is unknown, but may relate to the unique maternal immunology during pregnancy. Our findings were consistent with previous reports which suggest that EPA may be more effective than DHA in reducing inflammatory biomarkers [18].

By contrast, we found that fish oil supplementation in doses of approximately 1 g/day had no significant effect on umbilical cord blood cytokine concentrations. Our findings are not consistent with a systematic review of prior prenatal supplementation trials that found significant reduction of IL13 in infants of mothers who took prenatal fish oil supplementation [19]. This finding is also in contrast to our previously reported secondary analysis of the effect of fish oil supplementation on chemokine profiles among participants in this trial. That analysis found that the EPA- and DHA-rich fish oil had no significant effect on maternal chemokine concentrations, but significantly influenced cord blood chemokine ratios [20]. The reason for this discrepancy is unknown but may be related to the number of cord blood samples with cytokine concentrations that fell below detectable limits.

Based on our findings, we conclude that the fatty acid content of the prenatal maternal diet may modulate the maternal immune response, although prenatal supplementation did not significantly affect cord blood cytokine profiles in our study.

Strengths of our study included blinded assessment of outcomes, randomized design, and intent-to-treat analyses. The study also had several limitations. These included the differing times of day at which maternal blood samples were procured, a result of study procedures being timed with clinical care. However, all maternal samples were drawn after a four hour fast with only low-fat meals allowed on the day of the blood draw.

Another limitation of our study is that many cytokine concentrations were below the limits of detection for the assay used. These results were consistent with the results from other studies analyzed in the same laboratory and all of the study samples were analyzed on the same date. Likewise, the sample size of our study was based on the primary outcome of the Beck Depression Inventory score at 6 weeks’ postpartum. Nevertheless, our post-hoc power analysis suggests that this secondary study had reasonable power to detect a treatment effect of the fish oil supplementation on cytokine concentrations.

Additionally, our study was also limited by its double-dummy design which meant that all subjects in the intervention groups received soy oil placebo capsules along with the EPA- and DHA- rich fish oil capsules. Finally, it should be noted that the subjects were selected based on predisposition to depression and may have not been representative of the general population of pregnant women.

## Conclusions

Our findings allow us to hypothesize that among women with perinatal depressive symptoms related to inflammation, prenatal EPA-rich fish oil supplementation may reduce plasma concentrations of inflammatory cytokines. Current research in depression is moving toward identification of a subset of depressed individuals in whom dysregulated cytokine production may play a role in pathogenesis. Future research should focus on the role of omega-3 fatty acids, in particular EPA, in modulation of the immune response in this population, and should work to elucidate the relationship of inflammatory cytokines with depressive symptomatology in these individuals.

## Abbreviations

DHA: Docosahexaenoic acid
EPA: Eicosapentaenoic acid
IFNγ: Interferon γ
IL-10: Interleukin 10
IL-12p70: Interleukin 12p70
IL-13: Interleukin 13
IL-15: Interleukin 15
IL-17: Interleukin 17
IL-1α: Interleukin 1α
IL-1β: Interleukin 1 β,
IL-2: Interleukin 2
IL-4: Interleukin 4
IL-5: Interleukin 5
IL-6: Interleukin 6
IL-8: Interleukin 8
MCP-1: Monocyte chemotactic protein
TH1: Type 1 T helper cells
TNFα: Tumor necrosis factor α
TNFβ: Tumor necrosis factor β
